# Hepatitis E in blood donors: investigation of the natural course of asymptomatic infection, Germany, 2011

**DOI:** 10.2807/1560-7917.ES.2016.21.35.30332

**Published:** 2016-09-01

**Authors:** Tanja Vollmer, Juergen Diekmann, Matthias Eberhardt, Cornelius Knabbe, Jens Dreier

**Affiliations:** 1 Institut für Laboratoriums- und Transfusionsmedizin, Herz- und Diabeteszentrum Nordrhein- Westfalen, Universitätsklinik der Ruhr-Universität Bochum, Bad Oeynhausen, Germany; 2 TMD Gesellschaft für transfusionsmedizinische Dienste mbH, Kassel, Germany

**Keywords:** Hepatitis E virus, progression, anti-HEV IgA, anti-HEV IgM, anti-HEV IgG, seroconversion

## Abstract

Asymptomatic hepatitis E virus (HEV) infections have been found in blood donors from various European countries, but the natural course is rarely specified. Here, we compared the progression of HEV viraemia, serostatus and liver-specific enzymes in 10 blood donors with clinically asymptomatic genotype 3 HEV infection, measuring HEV RNA concentrations, plasma concentrations of alanine/aspartate aminotransferase, glutamate dehydrogenase and bilirubin and anti-HEV IgA, IgM and IgG antibodies. RNA concentrations ranged from 77.2 to 2.19×10^5^ IU/mL, with viraemia lasting from less than 10 to 52 days. Donors showed a typical progression of a recent HEV infection but differed in the first detection of anti-HEV IgA, IgM and IgG and seropositivity of the antibody classes. The diagnostic window between HEV RNA detection and first occurrence of anti-HEV antibodies ranged from eight to 48 days, depending on the serological assay used. The progression of laboratory parameters of asymptomatic HEV infection was largely comparable to the progression of symptomatic HEV infection, but only four of 10 donors showed elevated liver-specific parameters. Our results help elucidate the risk of transfusion-associated HEV infection and provide a basis for development of screening strategies. The diagnostic window illustrates that infectious blood donors can be efficiently identified only by RNA screening.

## Introduction

The hepatitis E virus is a single-stranded RNA virus; there are currently four human pathogenic genotypes 1 to 4 [1]. Genotypes 1 and 2 are hyperendemic in developing countries, restricted to humans, and transmission occurs by the faecal-oral route [2,3]. In industrialised countries, genotypes 3 and 4 are responsible for sporadic cases of HEV infection. However, the incidence of non-travel-associated HEV infections has increased and hepatitis E is now recognised as an emerging and often undiagnosed disease [1,4,5]. The genetic similarity of strains isolated from humans and other mammalian species suggests zoonotic or food-borne transmission [6,7].

Hepatitis E presents asymptomatically or symptomatically. Symptomatic infection presents as an acute, mostly self-limiting hepatitis with clinical characteristics similar to hepatitis A [2]. Clinical manifestations of HEV infections caused by the different genotypes are indistinguishable. Genotype 3 and 4 patients are usually middle-aged and elderly men, whereas genotypes 1 and 2 also cause acute hepatitis in healthy children and adolescents [8]. The pathogenic impact of genotype 1 and 2 and genotype 3 and 4 differ considerably. HEV genotype 1 and 2 infections lead to a high mortality among pregnant women in developing countries (8–20% [9,10]) while no serious infections among pregnant women with genotypes 3 and 4 were described in industrialised countries. HEV genotype 3 and 4 infection proceed asymptomatically in immunocompetent individuals [8], but severe or fatal HEV infections have been observed in individuals with chronic liver disease [11,12], in transplant patients [13,14] and in immunosuppressed individuals [8]. Asymptomatic HEV infection has often been observed in blood donors [15-17], with reported prevalence rates of HEV RNA-positive donors of 1:2,848 (England [18]), 1:1,240 (Germany [17]) and 1:1,761 (the Netherlands [19]). 

The progression of viraemia and the serological course of anti-HEV antibodies during clinically apparent HEV infection is well characterised [2,20,21], but so far little is known about the progression of infection in asymptomatic individuals, in whom HEV infection usually remains undetected. Therefore, we conducted a prospective study to characterise the duration of viraemia, the antibody response (IgA, IgM and IgG), and the progression of liver-specific enzymes in 10 HEV genotype 3-infected German blood donors [17].

## Methods

### Specimens

From July to September 2011, a total of 16,125 individual German blood donors were routinely screened for the presence of HEV RNA by the Uni.Blutspendedienst Ostwestfalen-Lippe. Their geographical origins were North Rhine-Westphalia, Lower Saxony and Hesse; 57.5% (n = 9,271) were male, with a median age of 33 years (± 13; range: 18–72), and 42.5% were female (n = 6,867), with a median age of 32 years (± 13; range: 18–71) [17]. The screening recovered 13 HEV RNA-positive donors. Retrospectively, residual plasma samples of one donation preceding and several donations following the initial HEV RNA-positive donation, taken within a short time distance from each other, (Table 1) were available for 10 donors (D1 to D10, all male). The day of the detection of HEV RNA by PCR screening was defined as day 0, but HEV infection is most likely to have occurred before the beginning of our study period. This aspect limits the exact calculation of the diagnostic window between the detection of HEV RNA and anti-HEV antibodies. In addition, the period of detectability of antibodies may have started before the first positive sample and lasted beyond the last positive sample. To take this into account, we calculated two intervals of HEV-RNA positivity: Interval 1 started on the day of the first positive and ended on the day of the last positive sample, whereas interval 2 started at half of the interval between the last negative and first positive sample and lasted until half of the interval between the last positive and first negative sample. The duration of anti-HEV seropositivity was calculated according to interval 2.

**Table 1:** Hepatitis E virus RNA progression in blood donors, Germany, 2011 (n = 10)

Donor	Maximum concentration (IU/mL)	Day^a^ with maximum concentration	Maximum concentration in window period (IU/mL)	Distance to last negative sample (days)	Distance to last positive sample (days)	Mean time between serial samples in days (range)	Duration interval 1^b^ (days)	Duration interval 2^c^ (days)
D1	2.63 × 10^4^	0	5.13 × 10^3^	43	10	5 (3–10)	20	(47)
D2	1.02 × 10^5^	25	1.02 × 10^5^	46	26	11 (5–26)	52	(88)
D3	1.51 × 10^3^	0	No window period	30	8	8 (8)	1	20
D4	4.74 × 10^4^	28	4.74 × 10^4^	9	6	10 (6–15)	42	50
D5	1.86 × 10^1^	0	No window period	> 1 year	3	7 (3–11)	11	(195)
D6	1.63 × 10^4^	21	1.63 × 10^4^	7	7	7 (7)	35	42
D7	2.13 × 10^4^	33	2.13 × 10^4^	7	3	6 (3–12)	46	51
D8	2.19 × 10^5^	28	2.19 × 10^5^	28	7	6 (3–12)	52	80
D9	1.36 × 10^3^	7	1.36 × 10^3^	54	42	16 (3–42)	7	(55)
D10	2.48 × 10^3^	21	2.48 × 10^3^	129	38	21 (21)	21	(105)
Range	1.86 × 10^1^ – 2.19 × 10^5^	0–33	1.36 × 10^3^ – 2.19 × 10^5^	NC	NC	NC	1–52	20–80
Mean	4.38 × 10^4^	20	5.19 × 10^4^	NC	NC	NC	29	49
Median	1.88 × 10^4^	23	1.88 × 10^4^	NC	NC	NC	28	50

NC: not calculated.

^a^ Day x post detection of HEV RNA by PCR screening.

^b^ Duration interval 1: first positive to last positive sample.

^c^ Duration interval 2: starting at half of the interval between the last negative and the first positive sample and ending at half of the interval between the last positive and the first negative sample. Data in parenthesis were excluded from the calculation of mean and median values because the last hepatitis E virus RNA-negative samples went back more than 30 days.

All HEV-infected donors underwent pre-donation medical examination and negated current diseases or any known risk factors for viral infection. Post-donation questionnaires to elucidate risk factors for HEV infection were returned by six donors. The study protocol followed the ethical guidelines of the Ruhr University, Bochum, and was approved by the institutional review board. All donors provided informed consent.

### RNA extraction and real-time RT-PCR

Total RNA from individual samples was extracted from 500 µl plasma using the NucliSens easyMAG (bioMerieux, Nürtingen, Germany) automated RNA/DNA extraction system. Amplification using the RealStar HEV RT-PCR Kit (Altona Diagnostic Technologies (ADT), Hamburg, Germany) was performed on the Rotor-Gene 3000 system (Corbett Life Sciences, Sydney, Australia). HEV virus titre in positive plasma was quantified using the first World Health Organization (WHO) international standard for hepatitis E virus RNA for NAT-based assays (Paul-Ehrlich institute, Langen, Germany) [22].

### Serological testing and measurement of liver-specific parameters

All plasma samples were screened for the presence of HEV-specific antibodies using the recomWell HEV IgM and recomWell IgG immunoassays (quantitative, Mikrogen GmbH, Neuried, Germany) and the Anti-HEV-IgA-ELISA (qualitative, Euroimmun, Lübeck, Germany). Analyses and serostatus interpretation were performed according to the manufacturers’ recommendations, results were classified into three categories: (i) no antibodies detectable (< 20 U/mL: negative), (ii) evidence for the presence of antibodies (≤ 20 to ≤ 24 U/mL: borderline) and (iii) antibodies detectable (> 24 U/mL: positive). Results (as the ratio extinction sample/calibrator) of the Anti-HEV-IgA-ELISA were classified as follows: (i) no antibodies detectable (ratio < 0.8: negative), (ii) evidence for the presence of antibodies (ratio > 0.8 to ≤ 1.1: borderline) and (iii) antibodies detectable (ratio > 1.1: positive).

Comparative testing was performed using the Wantai HEV IgM and IgG ELISA  (Sanbio B.V., Uden, the Netherlands), and results were classified into three categories: (i) no antibodies detectable (cut-off < 0.9: negative), (ii) evidence for the presence of antibodies (cut-off 0.9–1.1: borderline) and (iii) antibodies detectable (cut-off > 1.1: positive). Confirmatory testing with an immunoblot assay was performed on 22 samples using the recomLine HEV-IgM/IgG immunoassay according to the manufacturer’s instructions (Mikrogen GmbH, Neuried, Germany). Sample selection included those samples taken at the first positive detection of anti-HEV antibodies and up to two consecutive samples.

Concentrations of glutamate dehydrogenase (GLDH), aspartate aminotransferase (AST), alanine aminotransferase (ALT) and total bilirubin were measured in plasma samples using the respective enzymatic assays (Abbott Diagnostics Europe, Wiesbaden, Germany) on the Architect ci8200 system (Abbott Diagnostics Europe).

## Results

### Progression of hepatitis E virus RNA and anti-hepatitis E virus antibodies

The progression of RNA concentration in follow-up samples from infected patients is shown in Figure panel A, and the key observations of HEV RNA progression are summarised in Table 1. HEV viraemia persisted up to 52 days (D2 and D8, interval 1) with considerably different RNA concentrations in individual donors, ranging from 1.86×10^1^ to 2.19×10^5^ IU/mL. High RNA concentrations were observed in the window period ranging from 1.36×10^3^ to 2.19×10^5^ IU/mL. Taking the second interval into account, the duration of viraemia was as long as 20 to 80 days. The maximum viraemia was observed after 20 days, with a mean duration of 29 days for interval 1 and 49 days for interval 2 (Table 1).

**Figure:**
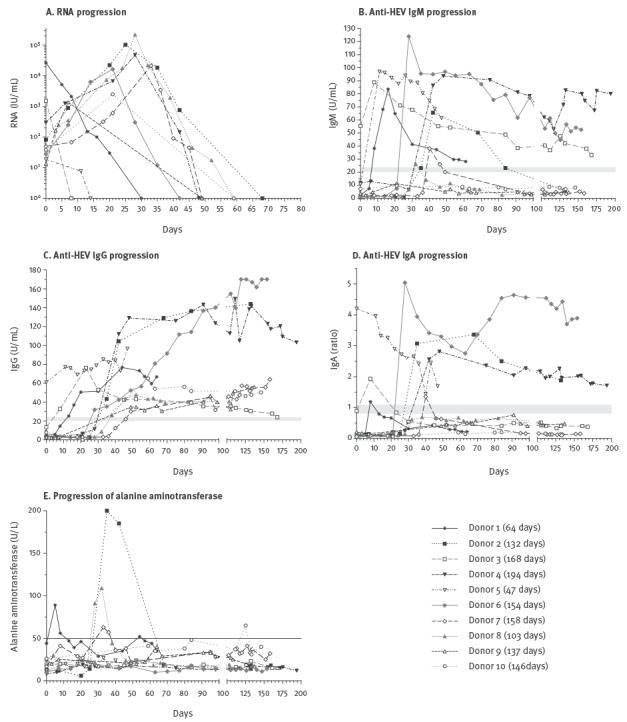
Progression of hepatitis E virus RNA, IgM, IgG and IgA antibodies and alanine aminotransferase in blood donors with autochthonous hepatitis E virus genotype 3 infection, Germany, 2011 (n = 10)

The day of the detection of HEV RNA by PCR screening was defined as day 0. Grey-shaded areas (panels B–D): cut-off values of the different serological assays as described in the Methods section. Solid horizontal line (panel E): reference range of 0–50 U/L. Days in brackets after donor legend: time period were samples were taken. Ratio: extinction sample/calibrator.

Figure panels B–D show the course of anti-HEV IgM, anti-HEV IgG (only results determined by the Mikrogen assay) and anti-HEV IgA. In samples of donor D3, HEV RNA and IgM antibodies were detectable in parallel. Likewise, HEV RNA, IgA and IgG antibodies were detected in parallel in samples of donor D5. This was probably due the fact that HEV infection occurred before the beginning of our HEV screening study period. The progression of anti-HEV IgA and IgM antibodies was virtually equal (Figure, panels B and D). Donor D8 did not have IgA antibodies at any time, and had a very limited increase of IgM antibodies that was only detectable on day 32 after the first detection of HEV RNA. In addition, IgA and IgM antibodies were not detectable in donors D9 and D10, but no samples were available between day 10 and 50 for donor D9 and between day 20 and 60 for donor D10, most probably including the time point where IgA/IgM seroconversion occurred. For the remaining donors, IgA, IgM and IgG antibodies were first detected between days 8 and 42 for IgA and IgM and between days 13 and 59 for IgG (Table 2, results Mikrogen assays).

**Table 2:** Occurrence of anti-hepatitis E virus antibody classes (IgA, IgM and IgG) and first detection and duration of IgM and IgG seropositivity in two serological assays, blood donors, Germany, 2011 (n = 10)

		IgA			IgM							IgG				
					Mikrogen	Wantai	Mikrogen	Wantai	Mikrogen	Wantai	Mikrogen	Mikrogen	Wantai	Mikrogen	Wantai	Mikrogen
Donor	Mean time between samples in days (range)	First positive detection on day x^a^	Duration^b^ and detection interval in days (range)	Maximum concentration on day x^a^	First positive detection on day x^a^	First positive detection on day x^a^	Duration^b^ and detection interval in days (range)	Duration^b^ and detection interval in days (range)	Maximum concentration on day x^a^	Maximum concentration on day x^a^	Maximum concentration (U/mL)	First positive detection on day x^a^	First positive detection on day x^a^	Maximum concentration on day x^a^	Maximum concentration on day x^a^	Maximum concentration (U/mL)
D1	6 (3–14)	8	5 (8)	8	8	8	> 55 (8 – >61)	> 55 (8– >61)	16	16	83.61	13	8	44	44	76.66
D2	17 (7–44)	35	102 (35 – >132)	68	35	35	> 54 (35 – >84)	> 54 (35 – >84)	42	42	65.55	35	35	132	132	143.9
D3	17 (7–44)	8	13 (8)	8	0	0	> 168 (0 – >168)	> 52 (0 – >52)	8	8	88.82	8	0	23	30	76.26
D4	11 (3–27)	42	159 (42 – >194)	48	42	42	159 (42 – >194)	80 (42–111)	48	48	93.62	42	42	111	130	149.39
D5	4 (3–11)	0	47 (0 – >47)	11	0	0	> 47 (0 – >47)	> 47 (0 – >47)	0	0	96.95	0	0	47	47	96.61
D6	7 (6–8)	28	130 (28– >154)	28	28	28	130 (28 – >154)	130 (28 – >154)	28	28	123.91	28	28	> 119	> 119	167.64
D7	8 (3–46)	40	6 (40)	40	40	40	10 (40–46)	34 (40 – >49)	40	40	38.07	49	40	158	158	63.96
D8	7 (3–21)	ND	ND	ND	32	28	7 (1)	23 (28–46)	32	32	26.23	39	39	52	52	47.74
D9	11 (3–21)	ND	ND	ND	ND	ND	ND	ND	ND	ND	ND	49	49	132	> 137	56.05
D10	19 (4–38)	ND	ND	ND	ND	ND	ND	ND	ND	ND	ND	59	59	59	59	65.03
Range	NC	8–42	5–159	8–68	8–42	8–42	7–159	23–130	16–48	16–48	26.23–123.9	13–59	8–42	44–158	30–158	47.74–167.64
Mean^c^	NC	31	80	38	31	30	69	63	34	34	71.83	34	32	103	89	108.20
Median^c^	NC	35	102	40	34	32	55	55	36	36	74.58	37	37	115	86	110.30

NC: not calculated; ND: no detection of antibodies.

^a^ Day x post detection of HEV RNA by PCR screening.

^b^ Duration: starting at half of the interval between the last negative and first positive sample and ending at half of the interval between last positive and first negative sample.

^c^ IgA: donors D3, D5, D8, D9 and D10 were excluded from the calculation of mean and median values. IgM/IgG: donors D3, D5, D9 and D10 were excluded from the calculation of mean and median values. Donors were excluded either because no antibodies were detected (IgA: D8, IgM/IgG: D9 and D10) or because all samples were taken ≥ 30 days from each other (D3 and D5).

In four donors (D1, D3, D7 and D8), IgM levels increased before IgG levels, and four donors (D2, D4, D5 and D6) showed a parallel increase of IgA, IgM and IgG. Detection of IgA before IgM was not observed, but IgA antibodies were detectable until the end of the observation period in donor D2 in the absence of IgM antibodies. In contrast, the detection period for anti-HEV IgM was longer than for IgA in donor D1 and donor D3. Three donors had detectable IgM (D3, D4 and D6) or IgA antibodies (D2, D4 and D6) more than 150 days after first detection of HEV RNA. The progression of IgG antibodies in donors D2 and D4 showed an almost equal rapid increase to high values of more than 100 U/mL 35 days after the first detection of HEV RNA (Figure, panel C). Donor D6 demonstrated a prolonged constant IgG increase to values higher than 100 U/mL, while the other donors showed a continuous moderate antibody increase (D1, D7, D8, D9 and D10) or a constant antibody titre (D5). A continuous decrease of anti-HEV IgG antibodies was observed in samples of donor D3.

Table 2 further summarises the key observations on the progression of anti-HEV IgA, IgM and IgG. Here we concentrate on the Mikrogen anti-HEV IgM/IgG results; the Wantai results will be described further down. The diagnostic window before the detection of HEV-specific antibodies was up to 42 days for IgA and IgM (D4) and up to 59 days for IgG (D10); the mean values including all donors were 31 days for IgA and IgM and 34 days for IgG. The mean duration of seropositivity was 80 days for IgA antibodies and 69 days for IgM antibodies. The maximum IgM and IgG titres differed considerably between different donors (IgM mean: 71.83 U/mL, range: 26.23–123.9; IgG mean: 108.20 U/mL, range: 47.74–167.64).

### Progression of liver specific enzymes

Elevated values of ALT were observed only for five donors (D1, D2, D7, D8 and D10). The ALT values showed a two- to fourfold (D1, D7, D8, D10) and an 11-fold (D2) increase compared with the reference value of 50 U/L. In donor D1, ALT levels showed two peaks, first on day 5, in the period when HEV RNA was detectable, and a second minor peak on day 55 in the absence of detectable HEV RNA. The three donors D2, D7 and D8 had elevated ALT values within the first 40 days after first HEV-RNA detection, with HEV RNA detected at the same time. AST and GLDH values followed the progression of ALT in these three donors, all other donors had normal AST and GLDH values (Figure and data not shown). Total bilirubin was within the reference range for all donors (data not shown).

### Comparison of the diagnostic window using different serological assays

We further compared the timing of the first detection of different antibody classes during the window period when only HEV RNA was detectable and the duration of seropositivity of HEV-specific IgM and IgG antibodies using two different serological assays (Table 2). For IgM and IgG antibodies, the diagnostic window differed depending on the assay used (Table 2), with a mean of 31 days (IgM) and 34 days (IgG) for the Mikrogen assay and a mean of 30 days (IgM) and 32 days (IgG) for the Wantai assay. In addition, the duration of IgM seropositivity depended on the serological assay: the Mikrogen assay had a longer detection period than the Wantai assay (mean: 69 days and 63 days, respectively) with a range of with 23 to 130 days (Wantai) vs seven to 159 days (Mikrogen). Overall, the Wantai assay showed a higher sensitivity than the Mikrogen assay and often detected IgM or IgG seropositivity at least one sampling point earlier (IgM: D8, IgG: D1, D3 and D7, Table 2). 

Samples taken at the first positive IgM and/or IgG detection by the two different assays and up to two consecutive samples were further analysed by immunoblot (Table 3). Borderline results were counted as positive. The Mikrogen ELISA, Wantai ELISA and immunoblot revealed concordant IgM results for 12 samples and concordant IgG results for 15 samples. For two IgM and two IgG samples, only the Wantai ELISA gave positive results. In eight IgM samples and five IgG samples, both ELISAs gave positive results but the interpretation of the immunoblot was negative.

**Table 3:** Hepatitis E virus-specific antigens in selected samples with different detection of anti-hepatitis E virus antibodies, Wantai vs Mikrogen ELISA, blood donors, Germany, 2011 (n = 8)

Donor (sex, age in years)	Day	Anti-HEV	Mikrogen	Wantai	Immunoblot^a^							
					O2N (1)		O2C (4)		O2M (1)	O3 (2)		∑ (interpretation)
					Gt1	Gt3	Gt1	Gt3		Gt1	Gt3	
D1 (M, 27)	5	IgM	Negative	Negative	−	−	−	−	−	−	−	0 (negative)
		IgG	Negative	Negative	−	−	−	−	−	−	−	0 (negative)
	13	IgM	Positive	Positive	+/−	+	+	−	−	+/−	+/−	5 (positive)
		IgG	Positive	Positive	−	−	+/−	+/−	−	−	−	0 (negative)
D2 (M, 37)	35	IgM	Borderline	Positive	+++	++	+/−	+/−	−	−	−	1 (negative)
		IgG	Positive	Positive	+	+/−	−	+	−	+++	++	7 (positive)
D3 (M, 26)	0	IgM	Positive	Positive	+/−	+	+	+/−	−	−	−	5 (positive)
		IgG	Negative	Positive	−	+/−	+/−	+/−	−	−	−	0 (negative)
	8	IgM	Positive	Positive	+/−	+	+	+/−	−	−	−	5 (positive)
		IgG	Positive	Positive	−	+/−	+/−	+	−	−	−	4 (positive)
D4 (M, 53)	42	IgM	Positive	Positive	−	−	−	+/−	−	−	−	0 (negative)
		IgG	Positive	Positive	−	−	+	+	−	+	+	6 (positive)
	48	IgM	Positive	Positive	−	−	−	+/−	−	−	−	0 (negative)
		IgG	Positive	Positive	−	−	+	+	−	+	+	6 (positive)
D5 (M, 26)	0	IgM	Positive	Positive	+/−	+/−	+	−	−	+	−	6 (positive)
		IgG	Positive	Positive	−	+	+	++	−	+++	+/−	7 (positive)
	11	IgM	Positive	Positive	−	−	+/−	+/−	−	+/−	−	0 (negative)
		IgG	Positive	Positive	−	+	+	++	−	+++	+/−	7 (positive)
D6 (M, 27)	21	IgM	Negative	Negative	−	−	−	−	−	−	−	0 (negative)
		IgG	Negative	Negative	−	−	−	−	−	−	−	0 (negative)
	28	IgM	Positive	Positive	+/−	+/−	+	−	−	+++	+++	6 (positive)
		IgG	Positive	Positive	−	−	−	+/−	−	++	+++	2 (negative)
	35	IgM	Positive	Positive	+/−	+/−	+/−	+/−	−	+++	+++	2 (negative)
		IgG	Positive	Positive	+	+/−	+/−	+	−	++	+++	7 (positive)
D7 (M, 22)	40	IgM	Positive	Positive	+	+	+/−	+/−	−	+	−	3 (borderline)
		IgG	Negative	Positive	+	+/−	−	−	−	+/ −	−	1 (negative)
	46	IgM	Positive	Positive	+/−	+/−	−	−	−	+/ −	−	0 (negative)
		IgG	Borderline	Positive	++	−	−	+/−	−	−	−	1 (negative)
	49	IgM	Borderline	Positive	+/−	+/−	−	−	−	+/−	−	0 (negative)
		IgG	Positive	Positive	++	+/−	−	+/−	−	+/−	−	1 (negative)
D8 (M, 26)	32	IgM	Positive	Positive	−	+/−	+/−	−	−	−	−	0 (negative)
		IgG	Negative	Negative	+/−	+/−	−	−	−	−	−	0 (negative)
	39	IgM	Negative	Positive	−	+/−	+/−	+/−	−	−	−	0 (negative)
		IgG	Positive	Positive	+/−	+/−	+/−	+/−	−	+/−	−	0 (negative)
	46	IgM	Negative	Positive	−	−	−	−	−	−	−	0 (negative)
		IgG	Positive	Positive	+/−	+/−	+	++	−	+/−	−	4 (positive)
D9 (M, 21)	49	IgM	Negative	Negative	NT	NT	NT	NT	NT	NT	NT	NT
		IgG	Positive	Positive	−	+/−	+	+	−	−	+/−	4 (positive)
	52	IgM	Negative	Negative	NT	NT	NT	NT	NT	NT	NT	NT
		IgG	Positive	Positive	−	+/−	+	+	−	−	+/−	4 (positive)
D10 (M, 20)	59	IgM	Negative	Negative	+/−	+/−	−	−	−	−	−	0 (negative)
		IgG	Positive	Positive	++	+/−	+	++	−	−	−	5 (positive)
	63	IgM	Negative	Negative	−	−	−	−	−	−	−	0 (negative)
		IgG	Positive	Positive	++	+/−	+	++	−	−	−	5 (positive)

HEV: hepatitis E virus; M: male; NT: not tested.

^a^ O2N, O2C, O2M, O3 (Gt1/Gt3: genotype 1 and 3): highly purified recombinant HEV antigens provided by the manufacturer; numeric score in parenthesis. −: no reaction; +/−: very weak intensity (< cut-off); +: weak intensity (= cut-off); ++: strong intensity (> cut-off); +++: very strong intensity. Interpretation: ≤ 2: negative; 3: borderline; ≥ 4: positive; only reactions with intensities higher than + were included in the interpretation. Numeric scores of antigens were summed up for final interpretation: once for samples with +, ++ or +++, and only once per antigen if Gt1 and Gt3 or both reacted. Calculation example for sample D2 IgG: 1 × score 1 (O2N Gt3 positive) + 1 × score 4 (O2C Gt3 positive) + 1 × score 2 (O3 Gt1 and Gt3 positive)

## Discussion

HEV viraemia in symptomatic cases usually lasts from four to six weeks but can remain more than 100 days in some cases [23]. Liver enzyme values reach a peak about six weeks post exposure before decreasing towards normal levels by week 10 [20]. The typical serological course of an HEV infection shows an initial rise in short-lived anti-HEV IgM after three to four weeks that decline to baseline levels within three to six months, followed by an increase of IgG which remains detectable for up to 15 years [2,20,21]. However, the knowledge about the natural course of HEV infection in asymptomatic HEV-infected individuals is limited.

The clinically asymptomatic cases analysed in this study represent the preselection of apparently healthy individuals voluntarily donating blood and lacking physically detectable symptoms of infection. The retrospective character of this study limited the availability of consecutive samples from the same donor taken less than 30 days apart and the accuracy of the calculated durations (viraemia, seropositivity). The observed differences in the sensitivity of the serological assays further influenced the calculation of the diagnostic window. For example, it has been shown that the performance of anti-HEV IgG assays strongly influences the estimation of hepatitis E seroprevalence [24]. The progression of HEV RNA in a Japanese cohort of 15 patients with acute symptomatic hepatitis E was largely comparable with what we observed in our study [25]. In contrast to our results, anti-HEV IgA and IgM (first detection: day 8–42) and IgG antibodies (first detection: day 13–59) in the Japanese cohort were detectable in symptomatic cases in parallel to the presence of HEV RNA at first sampling [25], pointing towards an earlier onset of viraemia in the patients without symptoms. Accordingly, anti-HEV IgA and IgM remained detectable until the end of the observation period in symptomatic cases in the Japanese cohort while two different progressions were observed in the asymptomatic cases in our study. Antibodies in some asymptomatic cases showed the same persistence as in symptomatic cases, whereas antibody levels in other asymptomatic cases continuously decreased and reached undetectable levels. Furthermore, we observed IgM positivity for a significantly longer period compared with the Japanese cohort with seropositivity (longer than 100 days in D3, D4 and D6). However, these differences between symptomatic and asymptomatic cases could be related to the performance of the ELISAs used. There is no consensus on whether immunoblot assays (rather than ELISAs) are needed in order to detect anti-HEV antibodies accurately. The immunoblot results in our study did not add informative value; the immunoblot provided negative results for samples with divergent results in the two different ELISAs, most probably because of inferior sensitivity.

Unexpectedly, anti-HEV IgG antibodies declined under detectable levels in samples from donor D3. Previous studies have shown that the period when anti-HEV IgG remains detectable can vary individually from six months to 14 years, but HEV IgG antibodies have also been shown to disappear [26-28]. Remarkably, a rise in liver-specific enzymes was observed only in four of 10 asymptomatic individuals, although high viral loads were detected in plasma. The elevation of ALT may have been missed in donors D9 and D10 because of the long delay of 42 and 38 days between two samples, respectively, but for the other eight donors, samples within the first 50 days after detection of HEV viraemia were taken at average intervals of less than 10 days.

There is an ongoing debate about HEV genotype 3 and 4 infection and blood safety. Published reports of HEV infections transmitted by contaminated blood products [29,30] and of the detection of HEV genotypes 3 and 4 in plasma fractionation pools [31] and blood donors [15-17] suggest that transfusion transmission of HEV is probably not uncommon, with many undiagnosed subclinical infections [15,16]. In a recent study by Hewitt et al., transmission of HEV genotype 3 via contaminated blood was demonstrated in 42% of transfusion recipients [18]. The clinical course (asymptomatic, mild hepatitis or acute liver failure) and severity of HEV infection in transfusion recipients are variable, most probably depending on predisposition or immune status. The vast majority of HEV genotype 3 and 4 infections are most likely to result in an asymptomatic course [32] but, for instance, chronic manifestations of HEV genotype 3 infection in immunosuppressed persons can become important in industrialised countries [33]. Feray et al. concluded that transfusion of blood products not screened for HEV RNA is associated with the risk of chronic infection in immunocompromised patients [34]. Nevertheless, the clinical relevance of transfusion-associated HEV infection is insufficiently understood and more data are needed regarding the duration of viraemia, the infective dose, the role of anti-HEV in the recipient and the frequency of clinically apparent transfusion-transmitted HEV infection [35]. Our results on the progression of HEV viraemia illuminate at least one of these questions. To our knowledge, neither the length of HEV window periods nor the course of HEV viraemia during window periods in blood donors have been studied so far. The observed high level viraemia during window period infection could represent an underestimated risk of HEV transmission.

Post-donation questionnaires returned by six donors did not reveal a potential source of HEV infection. None of the infected donors had travelled within two months before the HEV-positive donation. The consumption of pork meat was described by five of the six donors. The number of returned questionnaires in our study is too small for a statistically significant analysis. We currently perform routine HEV blood donor screening and ask those with positive results to answer a questionnaire. 

## Conclusion

We observed a diagnostic gap between the detection of high viral loads and the detection of anti-HEV antibodies, independently of the antibody class (IgA, IgM or IgG), in our cohort of clinically asymptomatic HEV-infected blood donors. The progression of viraemia and anti-HEV immunoglobulins was comparable to symptomatic cases, but a rise in liver-specific enzymes was infrequent in our blood donor cohort. Asymptomatic HEV infection make NAT screening methods necessary to detect infection and avoid transfusion of contaminated blood donations. However, the majority of infections are transmitted via the zoonotic or food-borne route. It is therefore important to focus public health measures both on blood safety and also on other infection routes for patients at risk, including immunosuppressed patients. 

## References

[1] Adlhoch C Kaiser M Pauli G Koch J Meisel H. Indigenous hepatitis E virus infection of a plasma donor in Germany. Vox Sang. 2009;97(4):303-8. 10.1111/j.1423-0410.2009.01211.x 19555366

[2] Purcell RH Emerson SU. Hepatitis E: an emerging awareness of an old disease. J Hepatol. 2008;48(3):494-503. 10.1016/j.jhep.2007.12.008 18192058

[3] Guthmann JP Klovstad H Boccia D Hamid N Pinoges L Nizou JY A large outbreak of hepatitis E among a displaced population in Darfur, Sudan, 2004: the role of water treatment methods. Clin Infect Dis. 2006;42(12):1685-91. 10.1086/504321 16705572

[4] Dalton HR Kamar N Izopet J. Hepatitis E in developed countries: current status and future perspectives. Future Microbiol. 2014;9(12):1361-72. 10.2217/fmb.14.89 25517900

[5] Rutjes SA Lodder WJ Lodder-Verschoor F van den Berg HH Vennema H Duizer E Sources of hepatitis E virus genotype 3 in The Netherlands. Emerg Infect Dis. 2009;15(3):381-7. 10.3201/eid1503.071472 19239749 PMC2681103

[6] Krumbholz A Mohn U Lange J Motz M Wenzel JJ Jilg W Prevalence of hepatitis E virus-specific antibodies in humans with occupational exposure to pigs. Med Microbiol Immunol (Berl). 2012;201(2):239-44. 10.1007/s00430-011-0210-5 21773797

[7] Matsuda H Okada K Takahashi K Mishiro S. Severe hepatitis E virus infection after ingestion of uncooked liver from a wild boar. J Infect Dis. 2003;188(6):944. 10.1086/378074 12964128

[8] Kamar N Dalton HR Abravanel F Izopet J. Hepatitis E virus infection. Clin Microbiol Rev. 2014;27(1):116-38. 10.1128/CMR.00057-13 24396139 PMC3910910

[9] Boccia D Guthmann JP Klovstad H Hamid N Tatay M Ciglenecki I High mortality associated with an outbreak of hepatitis E among displaced persons in Darfur, Sudan. Clin Infect Dis. 2006;42(12):1679-84. 10.1086/504322 16705571

[10] Christensen PB Engle RE Hjort C Homburg KM Vach W Georgsen J Time trend of the prevalence of hepatitis E antibodies among farmers and blood donors: a potential zoonosis in Denmark. Clin Infect Dis. 2008;47(8):1026-31. 10.1086/591970 18781880 PMC2803052

[11] Dalton HR Hazeldine S Banks M Ijaz S Bendall R. Locally acquired hepatitis E in chronic liver disease. Lancet. 2007;369(9569):1260. 10.1016/S0140-6736(07)60595-9 17434400

[12] Péron JM Bureau C Poirson H Mansuy JM Alric L Selves J Fulminant liver failure from acute autochthonous hepatitis E in France: description of seven patients with acute hepatitis E and encephalopathy. J Viral Hepat. 2007;14(5):298-303. 10.1111/j.1365-2893.2007.00858.x 17439518

[13] Kamar N Selves J Mansuy JM Ouezzani L Péron JM Guitard J Hepatitis E virus and chronic hepatitis in organ-transplant recipients. N Engl J Med. 2008;358(8):811-7. 10.1056/NEJMoa0706992 18287603

[14] Legrand-Abravanel F Kamar N Sandres-Saune K Garrouste C Dubois M Mansuy JM Characteristics of autochthonous hepatitis E virus infection in solid-organ transplant recipients in France. J Infect Dis. 2010;202(6):835-44. 10.1086/655899 20695798

[15] Baylis SA Gärtner T Nick S Ovemyr J Blümel J. Occurrence of hepatitis E virus RNA in plasma donations from Sweden, Germany and the United States. Vox Sang. 2012;103(1):89-90. 10.1111/j.1423-0410.2011.01583.x 22220775

[16] Ijaz S Szypulska R Tettmar KI Kitchen A Tedder RS. Detection of hepatitis E virus RNA in plasma mini-pools from blood donors in England. Vox Sang. 2012;102(3):272. 10.1111/j.1423-0410.2011.01554.x 21957873

[17] Vollmer T Diekmann J Johne R Eberhardt M Knabbe C Dreier J. Novel approach for detection of hepatitis E virus infection in German blood donors. J Clin Microbiol. 2012;50(8):2708-13. 10.1128/JCM.01119-12 22675127 PMC3421526

[18] Hewitt PE Ijaz S Brailsford SR Brett R Dicks S Haywood B Hepatitis E virus in blood components: a prevalence and transmission study in southeast England. Lancet. 2014;384(9956):1766-73. 10.1016/S0140-6736(14)61034-5 25078306

[19] Hogema BM Molier M Slot E Zaaijer HL. Past and present of hepatitis E in the Netherlands. Transfusion. 2014;54(12):3092-6. 10.1111/trf.12733 24889277 PMC4280434

[20] Dalton HR Bendall R Ijaz S Banks M. Hepatitis E: an emerging infection in developed countries. Lancet Infect Dis. 2008;8(11):698-709. 10.1016/S1473-3099(08)70255-X 18992406

[21] Kamar N Bendall R Legrand-Abravanel F Xia NS Ijaz S Izopet J Hepatitis E. Lancet. 2012;379(9835):2477-88. 10.1016/S0140-6736(11)61849-7 22549046

[22] Baylis SA Blümel J Mizusawa S Matsubayashi K Sakata H Okada Y World Health Organization International Standard to harmonize assays for detection of hepatitis E virus RNA. Emerg Infect Dis. 2013;19(5):729-35. 23647659 10.3201/eid1905.121845 PMC3647515

[23] Blood Advisory Committee. Topic I: Hepatitis E virus (HEV) and blood transfusion safety. 104th Meeting; 20 Sep 2012 Rockville; 2012. Available from: http://www.fda.gov/downloads/advisorycommittees/committeesmeetingmaterials/bloodvaccinesandotherbiologics/bloodproductsadvisorycommittee/ucm319542.pdf

[24] Wenzel JJ Preiss J Schemmerer M Huber B Jilg W. Test performance characteristics of Anti-HEV IgG assays strongly influence hepatitis E seroprevalence estimates. J Infect Dis. 2013;207(3):497-500. 10.1093/infdis/jis688 23148290

[25] Takahashi M Kusakai S Mizuo H Suzuki K Fujimura K Masuko K Simultaneous detection of immunoglobulin A (IgA) and IgM antibodies against hepatitis E virus (HEV) Is highly specific for diagnosis of acute HEV infection. J Clin Microbiol. 2005;43(1):49-56. 10.1128/JCM.43.1.49-56.2005 15634950 PMC540162

[26] Bendall R Ellis V Ijaz S Thurairajah P Dalton HR. Serological response to hepatitis E virus genotype 3 infection: IgG quantitation, avidity, and IgM response. J Med Virol. 2008;80(1):95-101. 10.1002/jmv.21033 18041018

[27] Khuroo MS Kamili S Dar MY Moecklii R Jameel S. Hepatitis E and long-term antibody status. Lancet. 1993;341(8856):1355. 10.1016/0140-6736(93)90873-F 8098491

[28] Mansuy JM Peron JM Bureau C Alric L Vinel JP Izopet J. Immunologically silent autochthonous acute hepatitis E virus infection in France. J Clin Microbiol. 2004;42(2):912-3. 10.1128/JCM.42.2.912-913.2004 14766888 PMC344494

[29] Dreier J Juhl D. Autochthonous hepatitis e virus infections: a new transfusion-associated risk? Transfus Med Hemother. 2014;41(1):29-39. 10.1159/000357098 24659945 PMC3949615

[30] Huzly D Umhau M Bettinger D Cathomen T Emmerich F Hasselblatt P Transfusion-transmitted hepatitis E in Germany, 2013. Euro Surveill. 2014;19(21):20812. 10.2807/1560-7917.ES2014.19.21.20812 24906377

[31] Baylis SA Koc O Nick S Blümel J. Widespread distribution of hepatitis E virus in plasma fractionation pools. Vox Sang. 2012;102(2):182-3. 10.1111/j.1423-0410.2011.01527.x 21806631

[32] Beale MA Tettmar K Szypulska R Tedder RS Ijaz S. Is there evidence of recent hepatitis E virus infection in English and North Welsh blood donors? Vox Sang. 2011;100(3):340-2. 10.1111/j.1423-0410.2010.01412.x 21392024

[33] Pischke S Behrendt P Bock CT Jilg W Manns MP Wedemeyer H. Hepatitis E in Germany--an under-reported infectious disease. Dtsch Arztebl Int. 2014;111(35-36):577-83. 25249359 10.3238/arztebl.2014.0577 PMC4174681

[34] Féray C Pawlotsky JM Roque-Afonso AM Samuel D Dhumeaux D. Should we screen blood products for hepatitis E virus RNA? Lancet. 2014;383(9913):218. 10.1016/S0140-6736(14)60072-6 24439737

[35] Juhl D Baylis SA Blümel J Görg S Hennig H. Seroprevalence and incidence of hepatitis E virus infection in German blood donors. Transfusion. 2014;54(1):49-56. 10.1111/trf.12121 23441647

